# Supplementary material for: Neural G0: a quiescent‐like state found in neuroepithelial‐derived cells and glioma
Source: Mol Syst Biol. 2021 Jun 8;17(6):e9522. doi: 10.15252/msb.20209522 (PMC8186478; doi:10.15252/msb.20209522)
Supplement: Supplementary file 1 — Appendix [file MSB-17-e9522-s005.docx]

Appendix to *Neural G0: a quiescent-like state found in neuroepithelial-derived cells and glioma*

Samantha A. O’Connor, Heather M. Feldman, Sonali Arora, Pia Hoellerbauer, Chad M. Toledo, Philip Corrin, Lucas Carter, Megan Kufeld, Hamid Bolouri, Ryan Basom, Jeffrey Delrow, José L. McFaline-Figueroa, Cole Trapnell, Steven M. Pollard, Anoop Patel, Patrick J. Paddison, and Christopher L. Plaisier

**Contents**

[1 Appendix Figures S1-20 3](#_Toc63357243)

[Appendix Figure S1 3](#_Toc63357244)

[Appendix Figure S2 5](#_Toc63357245)

[Appendix Figure S3 6](#_Toc63357246)

[Appendix Figure S4 7](#_Toc63357247)

[Appendix Figure S5 8](#_Toc63357248)

[Appendix Figure S6 9](#_Toc63357249)

[Appendix Figure S7 10](#_Toc63357250)

[Appendix Figure S8 11](#_Toc63357251)

[Appendix Figure S9 13](#_Toc63357252)

[Appendix Figure S10 14](#_Toc63357253)

[Appendix Figure S11 15](#_Toc63357254)

[Appendix Figure S12 17](#_Toc63357255)

[Appendix Figure S13 19](#_Toc63357256)

[Appendix Figure S14 21](#_Toc63357257)

[Appendix Figure S15 22](#_Toc63357258)

[Appendix Figure S16 24](#_Toc63357259)

[Appendix Figure S17 26](#_Toc63357260)

[Appendix Figure S18 28](#_Toc63357261)

[Appendix Figure S19 30](#_Toc63357262)

[Appendix Figure S20 31](#_Toc63357263)

[2 References 33](#_Toc63357264)

# **1 Appendix Figures S1-20**

## **Appendix Figure S1**


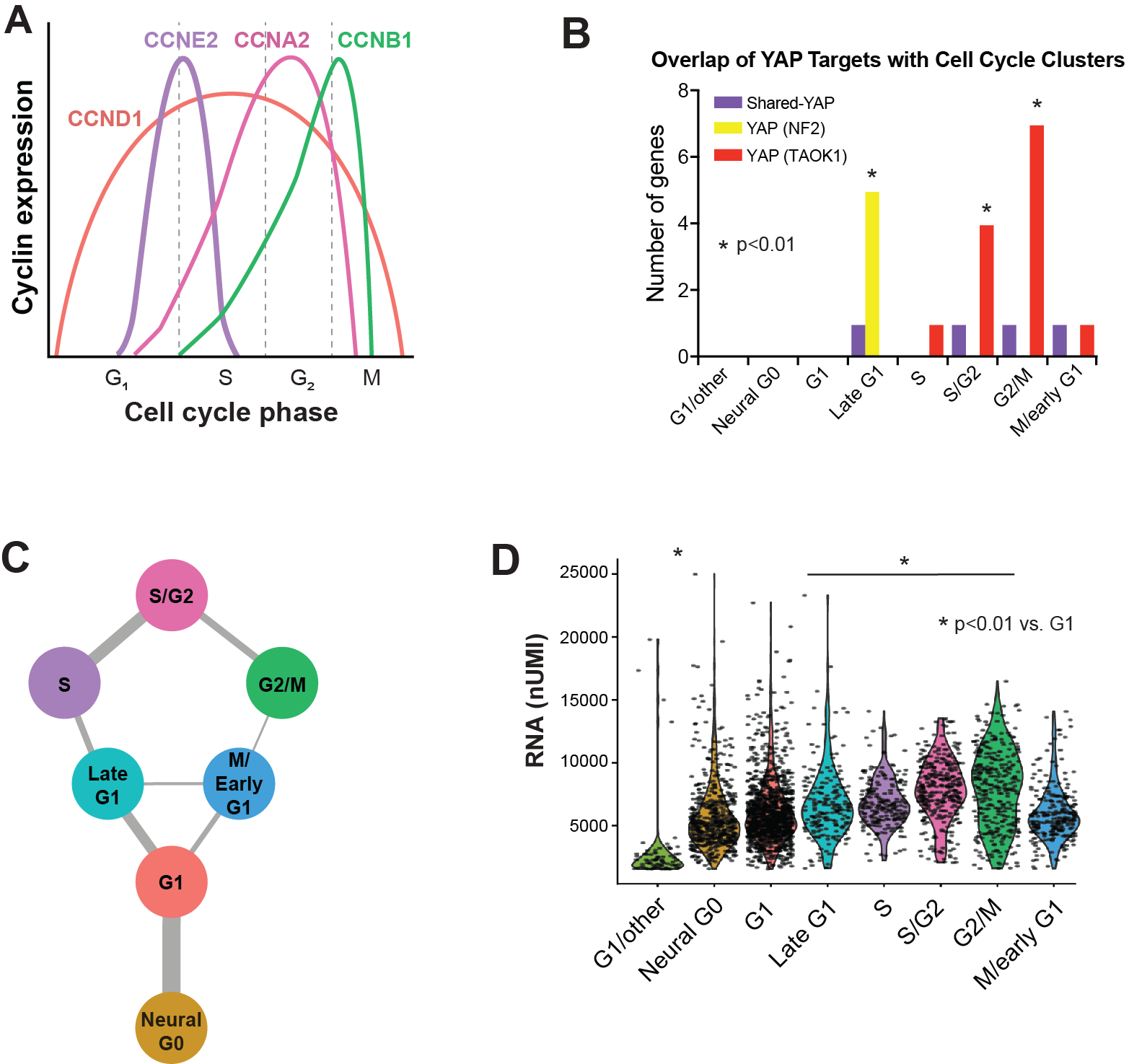


**Appendix Figure S1: Data in support of scRNA-seq data presented in Figure 1.**

**A**, Cyclin expression patterns are based upon Figure 1 from Darzynkiewicz et al, 1996 where flow cytometry coupled with intracellular cyclin antibody staining was performed. We slightly modified the CCND1 expression based on Figure 4 from Matsushime et al, 1994, which Darzynkiewicz et al, 1996 show in Figure 6 is a known expression pattern.

**B**, Overlap of conserved YAP target genes up-regulated following G0-skip knockout of *NF2* (yellow), *TAOK1* (red), or both (purple) with genes defining cell cycle transcriptional clusters. The NF2-dependent YAP targets significantly overlap with the Late G1 cluster, while the TAOK1-dependent genes overlap with the S/G2 and G2/M clusters. Both *NF2* and *TAOK1* knockouts are analyzed as G0-skip genes further in Figure 7 and Appendix Figure S11-12. Significance assessed using hypergeometric analysis.

**C**, Network describes most likely connections between the cell cycle clusters and recapitulates the canonical cell cycle. Network is derived from Canberra distance between cluster medoids.

**D**, Distribution of total unique molecular identifiers (nUMI) per cell grouped by U5-hNSC cluster. The G1/other cluster has dramatically less nUMI than any other cluster, making it difficult to distinguish actual cells belonging to this cluster from background. Therefore, this cluster was excluded from further analysis. The nUMI increases as the cell cycle progresses and peaks with the largest nUMI in the G2/M cells. Significance was assessed using a two-tailed student’s t-test.

## **Appendix Figure S2**

**Appendix Figure S2: tSNE plots for the top marker genes for each cell cycle phase (Figure 1F) classified in U5-hNSCs.**

Single cells are colorized by specific marker gene expression. Marker gene evaluated is displayed on the top left corner of each plot.

## **Appendix Figure S3**


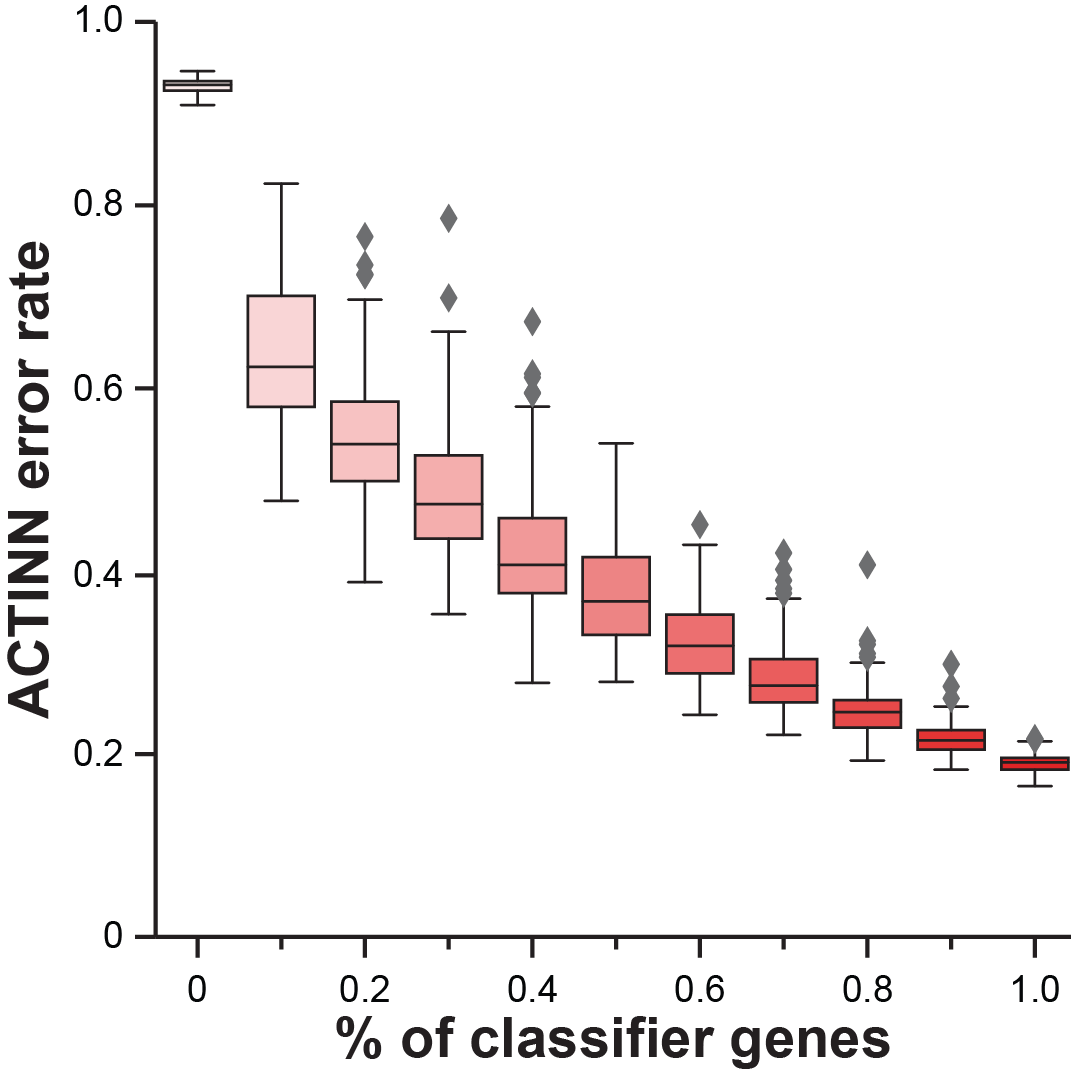


**Appendix Figure S3: Sensitivity analysis of ccAF ACTINN-based classifier with random sampling of a percentage of predictor genes.**

Sensitivity analysis shows how a random sampling of a percentage of the 1,536 input genes effects the error rate of the ccAF classifier.

## **Appendix Figure S4**

**Appendix Figure S4: Recreation of Figure 2 from Whitfield et al, 2002 with ccAF cell cycle phase classification.**

Figure 2 from Whitfield et al, 2002 uses gene expression for 20 well-characterized cell cycle genes across five different experimental cell cycle synchronization experiments (114 microarrays). We applied the ccAF classifier to the 1134 cell cycle genes that showed a cyclic pattern across the 114 time-series microarrays. There was an overlap of 199 genes with the classifier genes. The resulting ccAF cell cycle predictions for each time-point are shown as a track labeled “ccAF” above the gene expression. We also include the experimentally determined S and M phase as a track labeled as “Exp.” There was generally good agreement between the ccAF and experimental tracks (error rate = 13.7%).

## **Appendix Figure S5**

**Appendix Figure S5: Expression of Neural G0 marker genes in classifier across cells of the U5-hNSC study.**

Genes are rank ordered based on mean expression of genes from Neural G0-classified oRG cells from Nowakowski et al, 2017.

## **Appendix Figure S6**


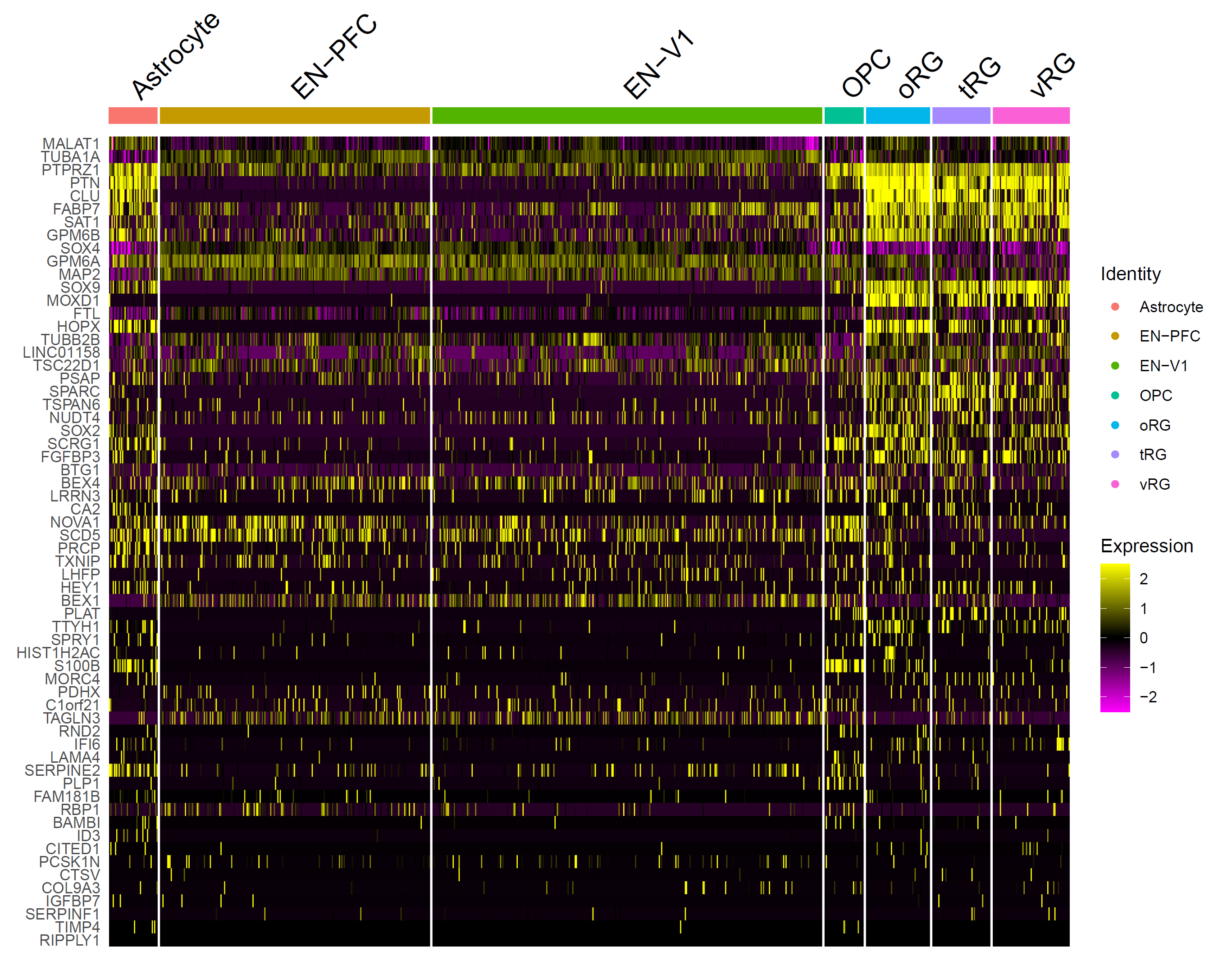


**Appendix Figure S6: Expression of Neural G0 marker genes in classifier across cells of the Nowakowski et al, 2017 study.**

Only the Astrocyte, EN-PFC, EN-V1, OPC, oRG, tRG, vRG cell types are shown. Genes are rank ordered based on mean expression of genes from Neural G0-classified oRG cells.

## **Appendix Figure S7**


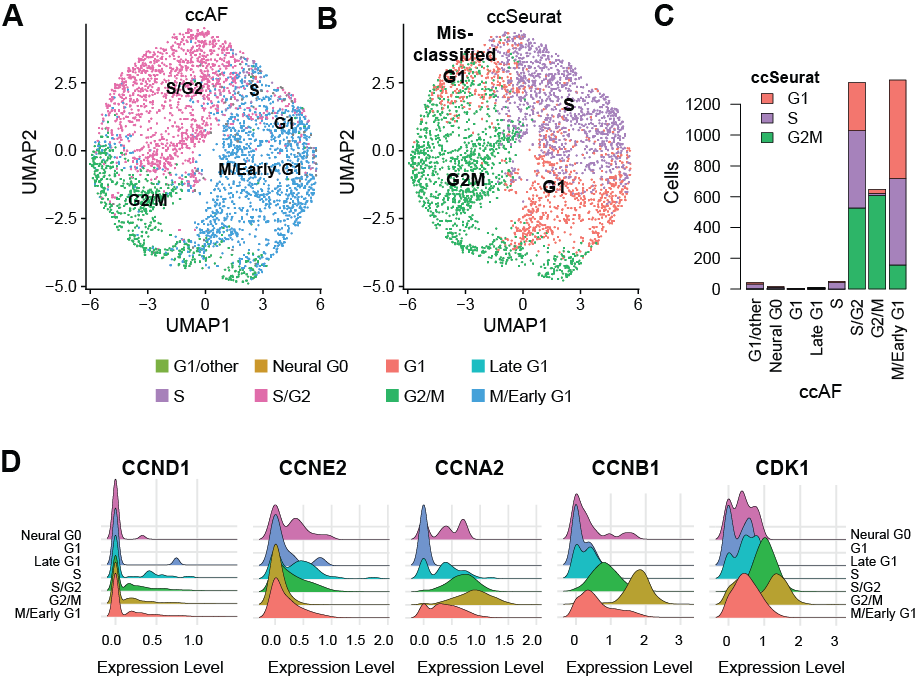


**Appendix Figure S7: Comparison of the ccAF and Seurat cell cycle classifiers with scRNA-seq data for HEK293T cells.**

**A,** UMAP plot of ccAF cell cycle classifier on scRNA-seq HEK293T data.

**B,** UMAP plot of Seurat cell cycle classifier on scRNA-seq HEK293T data**.**

**C**, Cell composition of Seurat cell cycle phase calls within ccAF-called cell populations.

**D,** Ridge graph comparisons of cyclin and CDK1 expression in ccAF-classified cell cycle phases.

## **Appendix Figure S8**

**
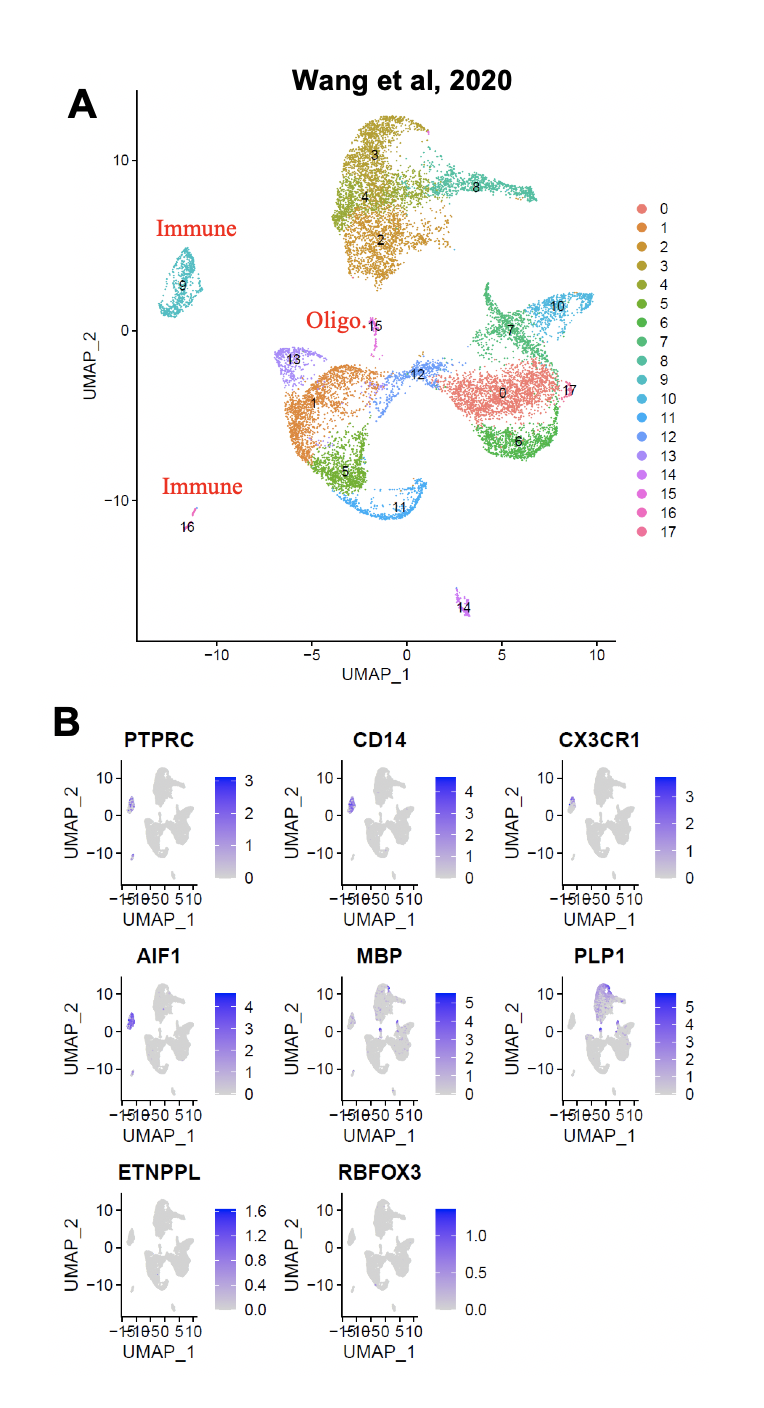
**

**Appendix Figure S8: Filtering glioma scRNA-seq to include only neoplastic cells, using Wang et al, 2020 (GSE139448) as an example.**

**A,** UMAP plots of all cells from Wang et al, 2020 overlaid with *de novo* clustering. Cluster 9 and 16 were determined to be immune based on expression of PTPRC, CD14, CX3CR1, and AIF1. Cluster 15 was determined to be oligodendrocytes based on expression of MBP and PLP1.

**B,** Feature maps with the same UMAP as in **A** with expression of the genes listed above overlaid as a heatmap.

## **Appendix Figure S9**

**Appendix Figure S9: Copy number alteration analysis was run on glioma scRNA-seq datasets using the inferCNV package. Darmanis et al, 2017 (GSE84465) is depicted here as an example.**

Top, Immune cell, OPC, Astrocyte, Oligodendrocyte, Vascular, and Neuron are listed as references. Bottom, Neoplastic cells from four patients are listed as observations. Cell types and patients are split according to color.

## **Appendix Figure S10**

**Appendix Figure S10: Dot plot to visualize the 22 Neural G0 marker genes’ expression across cell types in Darmanis et al, 2017.**

Each dot represents two values: marker gene mean expression within each cell type (visualized by color) and percentage of cells expressing marker gene in the cell type (visualized by dot size).

## **Appendix Figure S11**


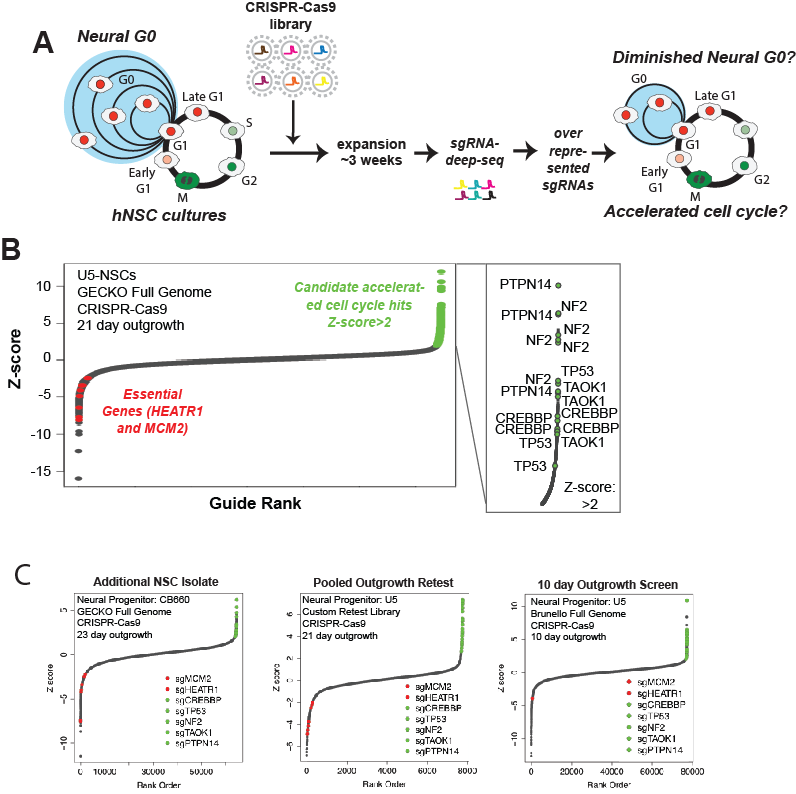


**Appendix Figure S11: CRISPR-Cas9 gene knockout screens to identify candidate Neural G0 regulating genes in hNSCs.**

**A,** Schematic of CRISPR-Cas9 knockout screen design.

**B,** Enrichment of guide DNA following 21 days outgrowth post-selection using GECKO library (n=2). Colored guides are enriched (z-score>2; green) or depleted (z-score<2; red) with false discovery rate (FDR<0.05). Statistical source data provided in Dataset EV7.

**C,** Enrichment of sgRNA using GECKO (n=2), Brunello (Doench *et al*, 2016) (n=3), or a custom-designed retest library (n=4), with U5-NSC or CB660-NSCs at 10 days or 3 weeks post-selection. Colored guides are enriched (z-score>2; green) or depleted (z-score<2; red) with false discovery rate (FDR)<0.05. Statistical source data provided in Dataset EV7.

## **Appendix Figure S12**


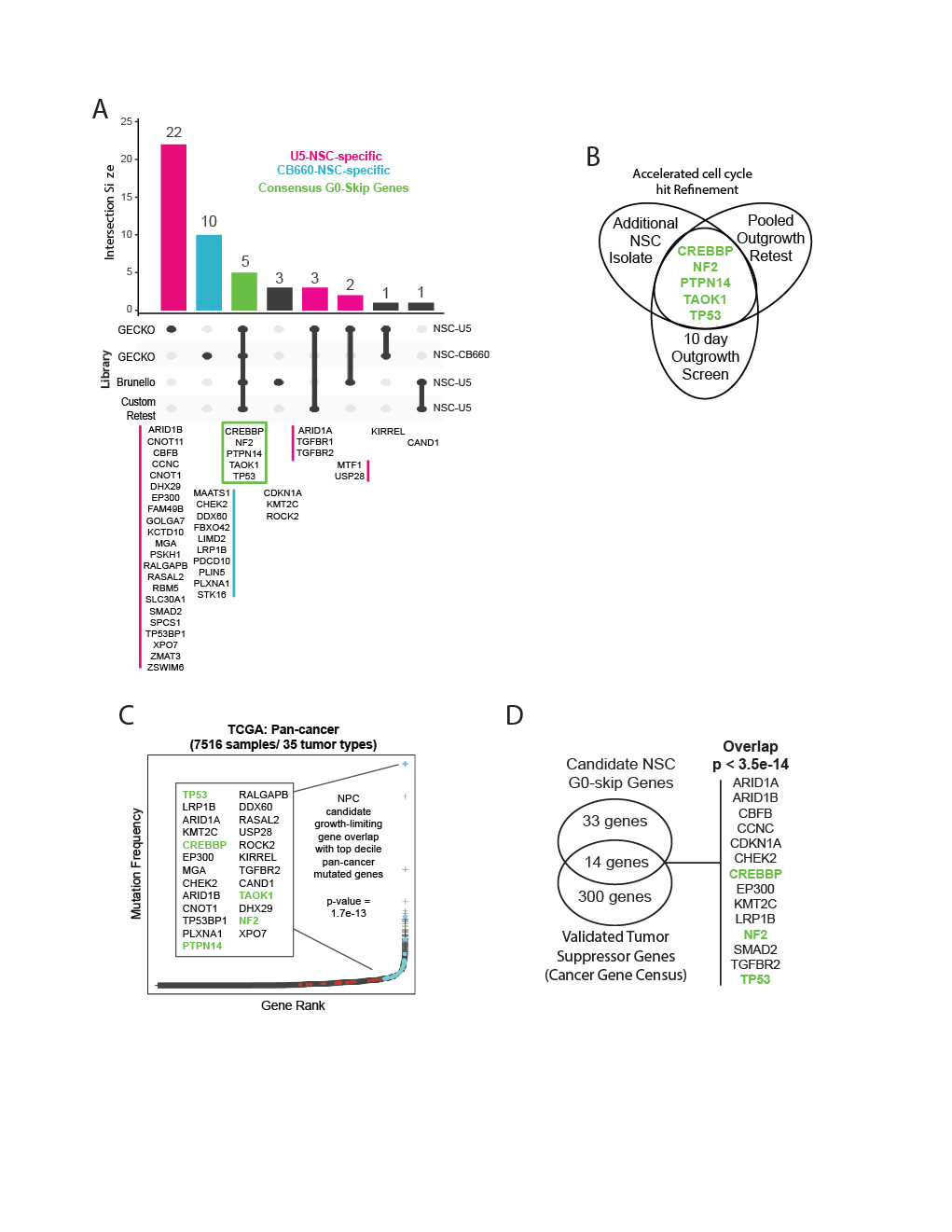


**Appendix Figure S12: Additional screens to identify Neural G0-skip mutants.**

**A**, Overlap of screen hits between the four CRISPR-Cas9 screens. For all screens, hits were defined as genes having multiple guides with FDR<.01 and z-score>2. For Brunello library, there were additional requirements of at least one guide with z-score>3 and at least 20 reads at Day 0. *CREBBP*, *NF2*, *PTPN14*, *TAOK1*, and *TP53* were defined as hits in every screen.

**B,** Overlap of screen hits between the four CRISPR-Cas9 screens. For all screens, hits were defined as genes having multiple guides with FDR<.01 and z-score >2. For Brunello library, there were additional requirements of at least one guide with z-score >3 and at least 20 reads at Day 0. *CREBBP*, *NF2*, *PTPN14*, *TAOK1*, and *TP53* were defined as hits in every screen.

**C**, Significant overlap between candidate growth-limiting genes and top decile pan-cancer mutated genes (The Cancer Genome Atlas (TCGA); hypergeometric analysis).

**D**, Overlap of the candidate antiproliferative genes with validated tumor suppressors (Futreal *et al,* 2004) (Tier 1 and 2; Cancer Gene Census v84). Significance assessed using hypergeometric analysis.

## **Appendix Figure S13**


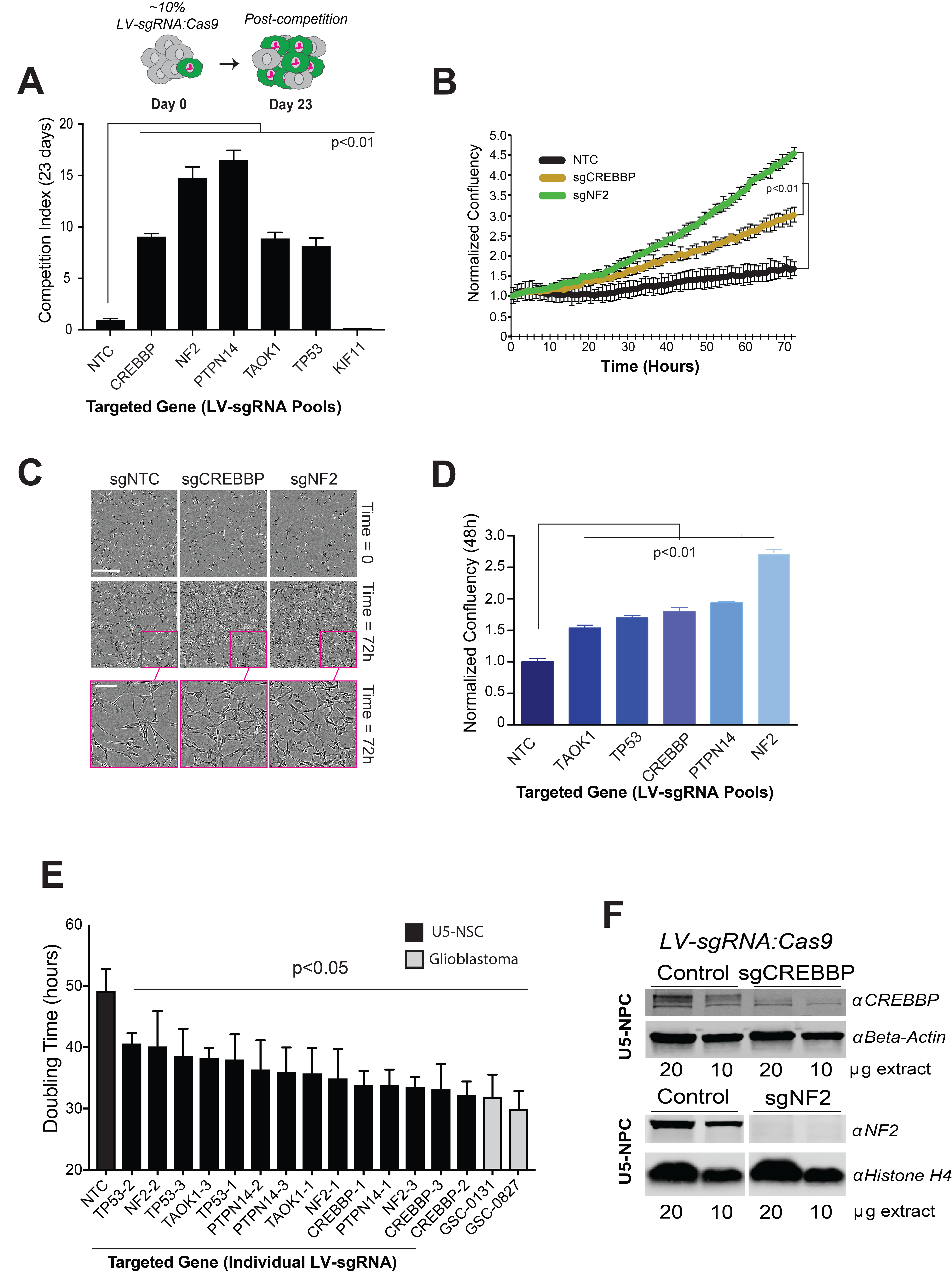


**Appendix Figure S13: Further retests and validation of G0-skip genes.**

**A**, Flow analysis of U5-NSC:GFP with LV-sgRNA:Cas9 retest pools competing with wild-type (WT) U5-NSC over a 23 day outgrowth with ~10% initial proportion (n=3). Competition index refers to the relative increase in %GFP+ compared with initial proportion and mean sgNTC.

**B,** Relative confluency of pooled sgCREBBP and sgNF2, and NTC in U5-NSCs compared to initial confluency over 72 hours at subconfluent density using time lapse microscopy (n=4 sampling regions x 3 biological replicates).

**C,** Representative images of cell confluency at time points 0 and 72h in LV-sgCREBBP and sgNF2 treated cells. Scale bars = 150 μm and 50 μm.

**D,** Confluency of cells relative to time=0 in comparison to NTC after 48 hours using time-lapse microscopy (n=4 sampling regions x 3 biological replicates).

**E**, Doubling time measurements (>14 days post-selection) in U5-NSCs or GSCs after 3-5 days outgrowth. n≥3, as noted in bars for each guide. The data are presented as the mean ± standard deviation (SD). Significance was assessed using a two-tailed student’s t-test.

**F**, Western blot confirmation of CREBBP and NF2 protein depletion after gene targeting using lentiviral (LV) pools (4 guides/gene) sgRNA:Cas9 after >21 days outgrowth.

## **Appendix Figure S14**


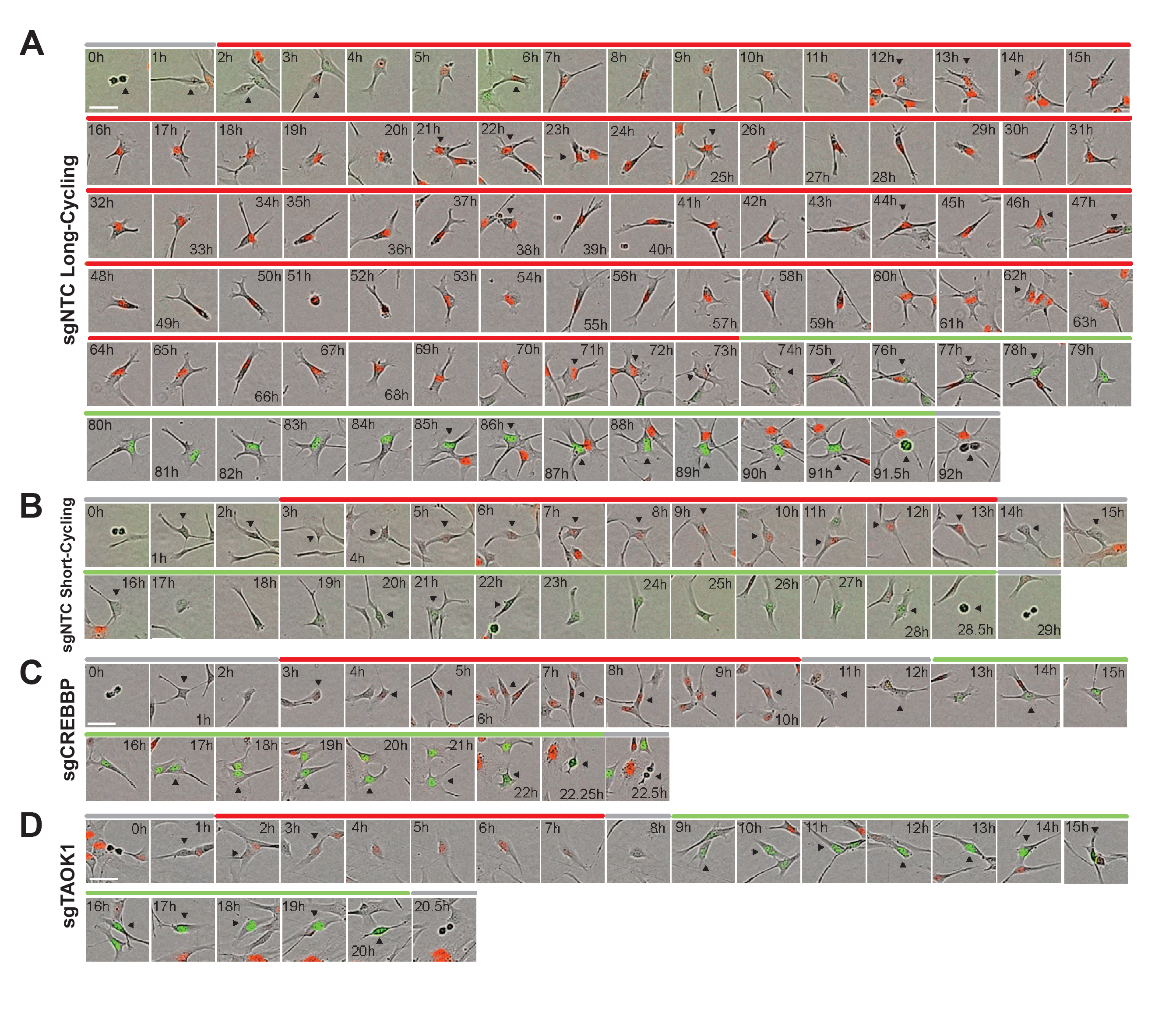


**Appendix Figure S14: Data supporting Figure 6D for time-lapse analysis of NSC cell cycle using FUCCI factors.**

**A-D**, The complete cell cycle of individual U5-NSCs expressing FUCCI factors that combine cell cycle-dependent degrons with fluorophores. mCherry-CDT1(aa30-120) (red) is present in G0/G1 and mAG-geminin(aa1-110) (green) is present in S/G2/M. The double negative cells correspond to either a transition state between M and G1 or G1 and S phase or cells that have silenced one or both reporters. Double positive cells which express both CDT1 and Geminin are an alternative G1-S transition state but are rare in the U5-NSC isolate. We tracked individual cells from mitosis to mitosis using timelapse microscopy, every 10-15 minutes, and representative cells from long-cycling NTC (A), short-cycling NTC (B), sgCREBBP (4 guide lentiviral pool) (C), and sgTAOK1 (4 guide lentiviral pool) (D) are presented here.

## **Appendix Figure S15**


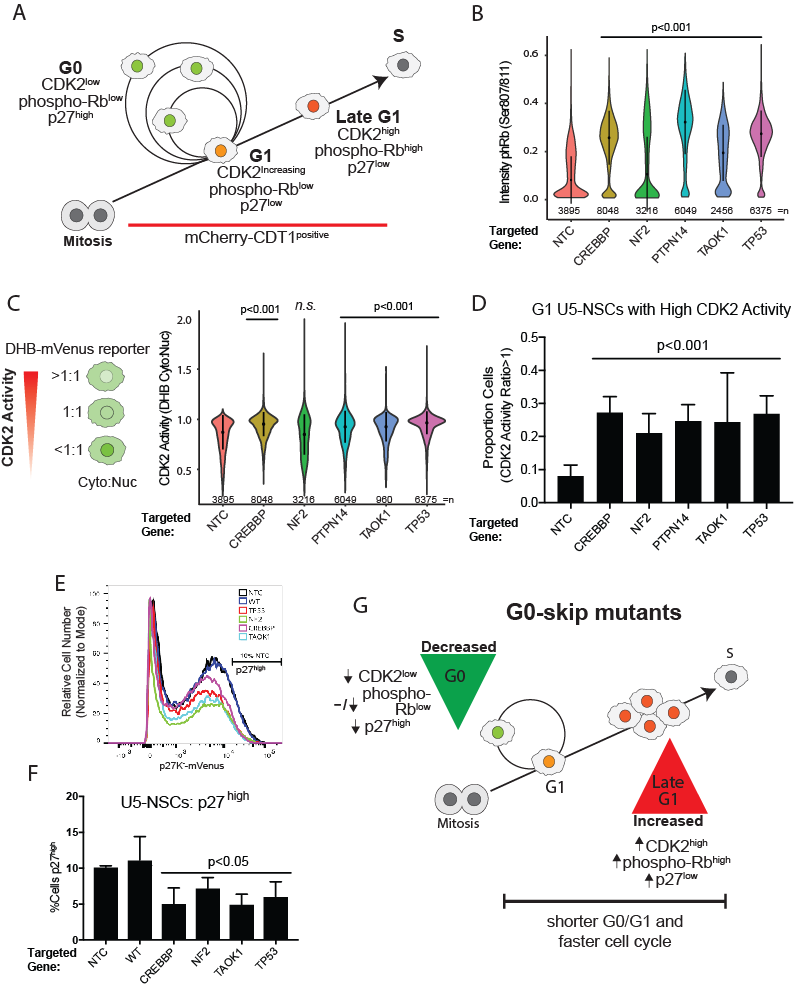


**Appendix Figure S15: Loss of G0*-*skip genes reduces G0 and increases Late G1 molecular features.**

**A,** Diagram of key molecular features for G0/G1 subpopulations.

**B,** Intensity of immunofluorescence staining for phosphorylated Rb (Ser807/811) in G0/G1 U5-NSCs (mCherry-CDT1+) (Median ± SD; n=2456-8048 cells).

**C,** CDK2 activity in G0/G1 hNSCs after KO measured through the relative ratio of a DNA helicase B (DHB) reporter (Hahn *et al*, 2009) using fluorescence microscopy. G0/G1 nuclei were identified by the presence of mCherry-CDT1 and due to the irregular shape of the NSCs, the cytoplasmic intensity of the DHB reporter was defined as the upper quartile intensity of a 2-pixel ring around the CDT1-defined nucleus (n=960-8048 cells). *NF2* KO was not significantly different from NTC (p=0.51).

**D,** Proportion of G0/G1 cells with cytoplasmic:nuclear ratio >1 for CDK2 activity reporter (n=54 fields).

**E,** Representative distribution of the entire population of U5-NSCs for p27K^-^-mVenus assayed using flow cytometry after KO along with WT and NTC*.* Threshold to define p27^high^ set at the top ~10% of the NTC for each independent replicate.

**F,** Proportion of cells p27^high^ following KO, assayed using flow cytometry (n=3).

**G,** Model for the changes in G0/G1 molecular features following loss of *CREBBP*, *NF2*, *PTPN14*, *TAOK1*, or *TP53*.

The data are presented as the mean ± SD. Significance was assessed using a two-tailed student’s t test.

## **Appendix Figure S16**


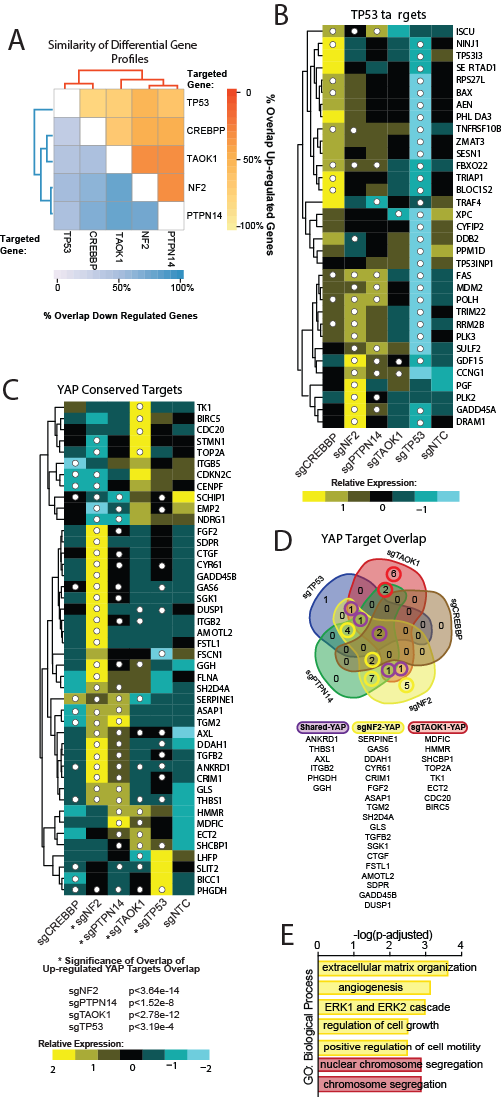


**Appendix Figure S16: Transcriptional target and cell cycle gene expression following loss of G0-skip genes.**

**A**, Hierarchical clustering and percent overlap of differentially expressed up- and down-regulated genes between G0-skip KO and NTC G0/G1 U5-NSCs. Percent gene overlap is relative to the smaller gene set.

**B**, Heat map of high-confidence TP53 target genes (direct regulation score ≥ 10) (Fischer, 2017) that are significantly changed (white dots, FDR<0.05) in at least one G0-skip KO compared to NTC in G0/G1 U5-NSCs measured through RNA-sequencing. The reduction of TP53-dependent transcription is only seen in the TP53 KO.

**C**, Heat map of conserved YAP target genes (Cordenonsi *et al,* 2011) that are significantly changed (white dots, FDR<0.05) in at least one G0-skip KO compared to NTC in G0/G1 U5-NSCs measured through RNA-sequencing. There are unique subsets of Hippo-YAP target genes up-regulated in *NF2* and *TAOK1* KOs. Significance of overlap assessed through hypergeometric analysis.

**D**, Venn diagram of YAP target overlap between G0/G1 U5-NSCs with G0-skip KO shows distinct gene sets up-regulated by NF2 (sgNF2-YAP) and TAOK1 (sgTAOK1-YAP) KOs as well as some shared targets. *PTPN14* KO is most similar to NF2 KO but shares some target up-regulation with *TAOK1* KO.

**E**, Gene ontology analysis of the sgNF2-YAP (yellow) and sgTAOK1-YAP (red) target subsets.

## **Appendix Figure S17**


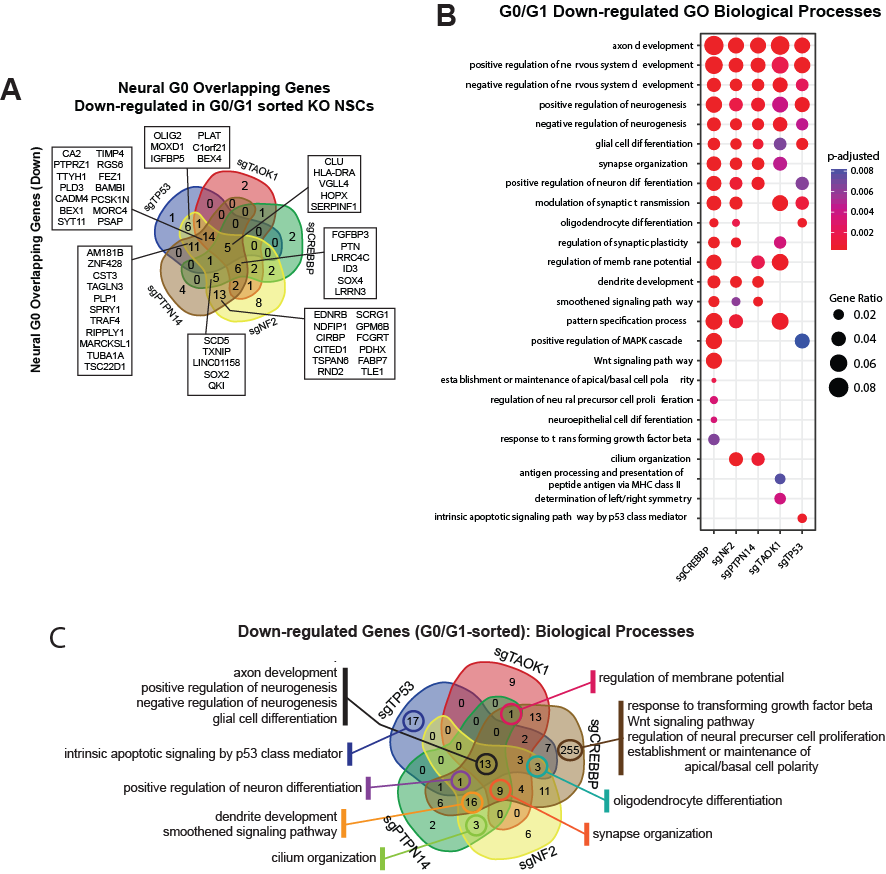


**Appendix Figure S17: Gene ontology analysis of genes down-regulated in G0/G1 following loss of G0-skip genes in hNSCs.**

**A**, Overlap of genes that are significantly down-regulated following G0-skip KO in G0/G1 U5-NSCs compared to the Neural G0 single cell RNA-sequencing cluster.

**B,** Dot plot of selected enriched gene ontology (GO) biological processes following G0-skip KO in G0/G1 U5-NSCs for up- (A) and down- (B) regulated genes compared to NTC. Gene ratio compares the proportion of genes contained within the gene ontology group that are significantly changed in the G0-skip KOs to the total number of genes significantly changed in the G0-skip mutants.

**C,** Overlap of a selection of biological processes enriched in down-regulated genes in G0/G1 following KO of G0-skip genes using gene ontology analysis. Full list in Supplementary Dataset EV7.

## **Appendix Figure S18**


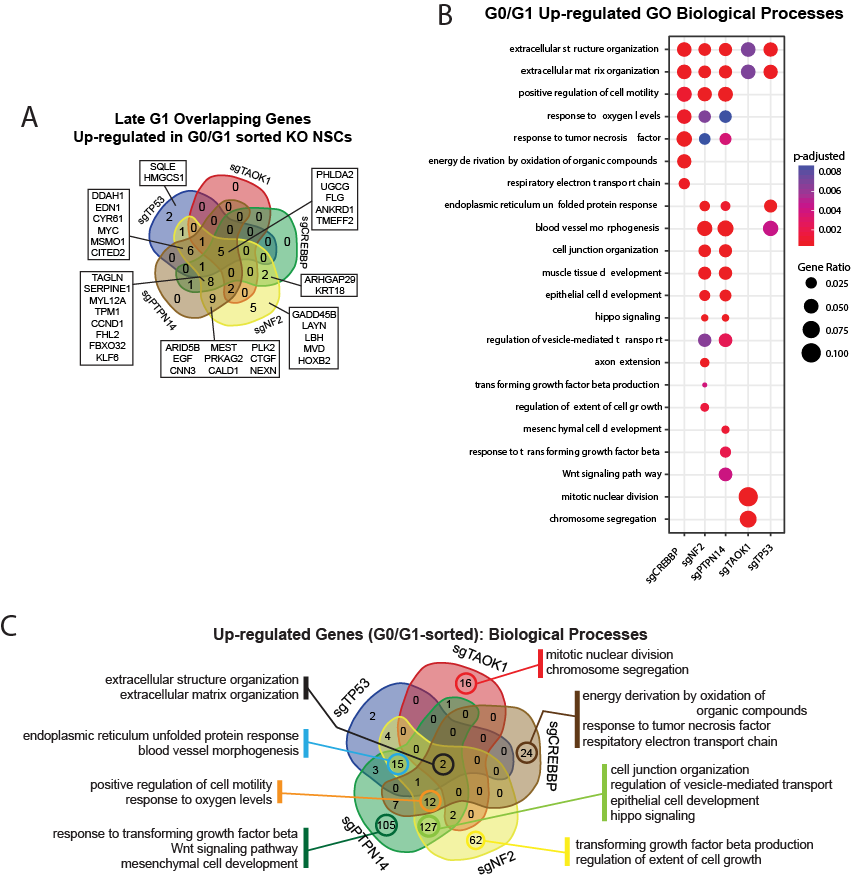


**Appendix Figure S18: Gene ontology analysis of genes up-regulated in G0/G1 following loss of G0-skip genes in hNSCs.**

**A**, Overlap of genes that are significantly up-regulated following G0-skip KO in G0/G1 U5-NSCs compared to the Late G1 (B) single cell RNA-sequencing cluster.

**B,** Dot plot of selected enriched gene ontology (GO) biological processes following G0-skip KO in G0/G1 U5-NSCs for up-regulated genes compared to NTC. Gene ratio com- pares the proportion of genes contained within the gene ontology group that are significantly changed in the G0-skip KOs to the total number of genes significantly changed in the G0-skip mutants.

**C,** Overlap of a selection of biological processes enriched in up-regulated genes in G0/G1 following KO of G0-skip genes using gene ontology analysis. Full list in Supplementary Dataset EV7.

## **Appendix Figure S19**


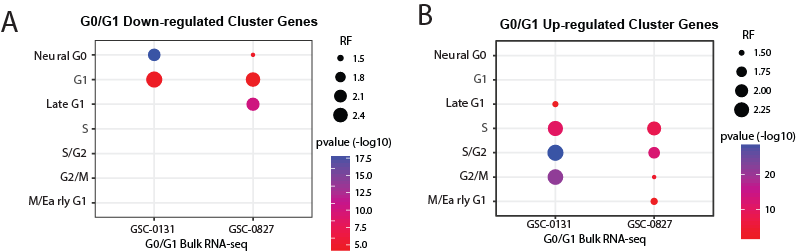


**Appendix Figure S19: hNSC cell cycle cluster genes altered in patient-derived GSCs.**

Significance of overlap of the down- (A) and up- (B) regulated genes from bulk RNA-sequencing of G0/G1 sorted GSC-0131 and GSC-0827 cells with the single cell cluster definitions (up-regulated genes). Significance assessed though hypergeometric analysis. RF = representation factor.

## **Appendix Figure S20**


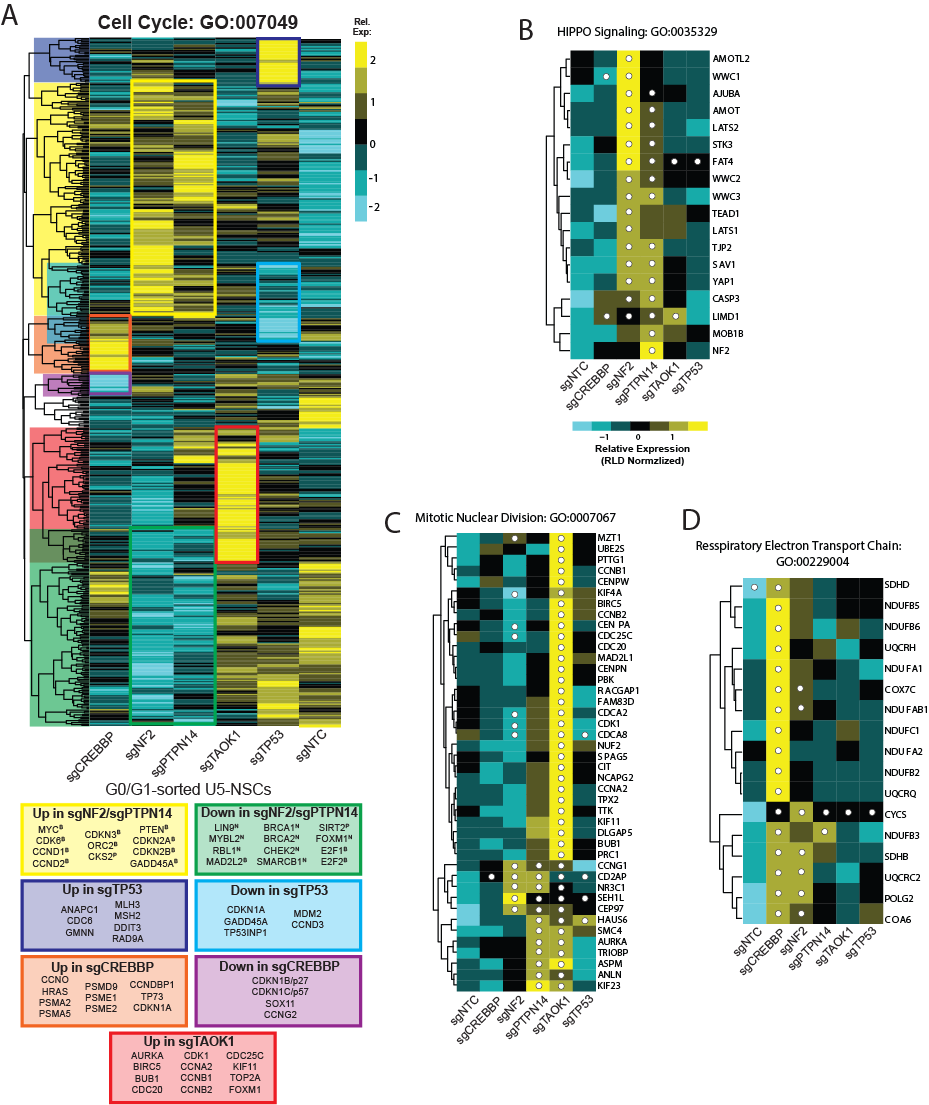


**Appendix Figure S20: Additional gene expression analysis of G0-skip mutants in hNSCs.**

**A-D**, Heat maps for genes (FDR<0.05) defining some of the significant biological process gene ontology groups in G0/G1 U5-NSCs (Dataset EV7). (A) Cell cycle genes significantly altered in each G0-skip mutant. (B) HIPPO signaling genes (GO:0035329), significantly enriched in *NF2* (p-adj.=3.52E-05) or *PTPN14* KO (p-adj.=9.42E-05). (C) Mitotic nuclear division genes (GO:007067) significantly enriched in *TAOK1* KO (p-adj.=3.16E-10). (D) Respiratory electron transport chain genes (GO:00229004) significantly enriched in *CREBBP* KO (p-adj.=1.62E-04). All heat maps were normalized to the regularized log (RLD). Scale applies to all heat maps and refers to the row-wise z-score. White dots indicate gene expression significantly different from NTC (FDR<0.05).

# **2 References**

Darzynkiewicz Z, Gong J, Juan G, Ardelt B, Traganos F. Cytometry of cyclin proteins. Cytometry. 1996;25(1):1-13. doi:10.1002/(SICI)1097-0320(19960901)25:1<1::AID-CYTO1>3.0.CO;2-N

Doench JG, Fusi N, Sullender M, Hegde M, Vaimberg EW, Donovan KF, Smith I, Tothova Z, Wilen C, Orchard R et al. 2016. Optimized sgRNA design to maximize activity and minimize off-target effects of CRISPR-Cas9. *Nat Biotechnol* **34**: 184-191.

Gao J, Aksoy BA, Dogrusoz U, Dresdner G, Gross B, Sumer SO, Sun Y, Jacobsen A, Sinha R, Larsson E et al. 2013. Integrative analysis of complex cancer genomics and clinical profiles using the cBioPortal. *Sci Signal* **6**: pl1.

Hahn AT, Jones JT, Meyer T. 2009. Quantitative analysis of cell cycle phase durations and PC12 differentiation using fluorescent biosensors. *Cell cycle* **8**: 1044-1052.

Matsushime H, Quelle DE, Shurtleff SA, Shibuya M, Sherr CJ, Kato JY. D-type cyclin-dependent kinase activity in mammalian cells. Mol Cell Biol. 1994;14(3):2066-2076. doi:10.1128/mcb.14.3.2066

Whitfield ML, Sherlock G, Saldanha AJ, Murray JI, Ball CA, Alexander KE, Matese JC, Perou CM, Hurt MM, Brown PO et al. 2002. Identification of genes periodically expressed in the human cell cycle and their expression in tumors. *Mol Biol Cell* **13**: 1977-2000.
